# Mycetoma Caused by Acremonium Species in a Patient with Chronic Granulomatous Disease

**DOI:** 10.1155/2016/3209493

**Published:** 2016-02-28

**Authors:** Richard Antrobus, Gabriel Wong, Julie Jones, Aarnoud Huissoon

**Affiliations:** Department of Immunology, Heartlands Hospital, Bordesley Green East, Birmingham B9 5SS, UK

## Abstract

Patients with chronic granulomatous disease are predisposed to fungal infections and are therefore routinely prescribed antifungal prophylaxis. We report a case where acremonium was responsible for causing a cutaneous infection (mycetoma) despite antifungal prophylaxis. Treatment with voriconazole was initiated and the infection gradually resolved. This case highlights the need for careful clinical follow-up and thorough investigation of patients who have a neutrophil immunodeficiency.

## 1. Introduction

Chronic granulomatous disease (CGD) is a primary immunodeficiency syndrome characterised by impaired neutrophil superoxide generation and microbial killing. It is a rare condition, with one affected case per 200,000–250,000 live births [1]. Autosomal recessive and X-linked forms occur with the latter accounting for approximately two-thirds of cases. In the X-linked form, mutations in the* CYBB* gene result in a loss of gp91phox function. As a result, NADPH oxidase activation is reduced and an important killing mechanism for neutrophils is defective.

CGD patients are susceptible to infection from a range of microorganisms, but bacteria and fungi predominate. The most common cause of severe fungal infections is aspergillus [2]. Of nonaspergillus fungal infections in CGD, acremonium contributes just less than 3% of the reported cases [3]. Here we report what we believe is the first occurrence of a cutaneous infection due to acremonium in a young adult with X-linked CGD.

## 2. Case Presentation

Our patient was diagnosed with CGD in his first year of life as a result of his older brother being diagnosed with the same condition. The diagnosis was made on the basis of a nitroblue-tetrazolium (NBT) test but not confirmed with molecular genetics. He had previously experienced episodes of pneumonia, colitis, perianal abscesses with fistulae, and other bacterial abscesses as complications of his CGD.

He presented at 20 years old with a subcutaneous swelling over the medial aspect of his left scapular. At this point his regular medications were itraconazole 200 mg and co-trimoxazole 960 mg (both once daily) which were prescribed for antimicrobial prophylaxis. Itraconazole levels at the time of presentation measured 0.51 mg/L which are normally deemed adequate for prophylaxis (but not treatment).

The swelling was fluctuant and mildly tender. An infective aetiology was suspected and therefore a diagnostic aspirate was performed. This yielded nonoffensive blood stained pus, and samples were sent for routine microscopy and culture. He was prescribed one week of empirical antibiotics (co-amoxiclav 625 mg tds and clindamycin 300 mg qds). In the laboratory, pus cells were demonstrated on microscopy but standard cultures were negative.

**Figure 1:**
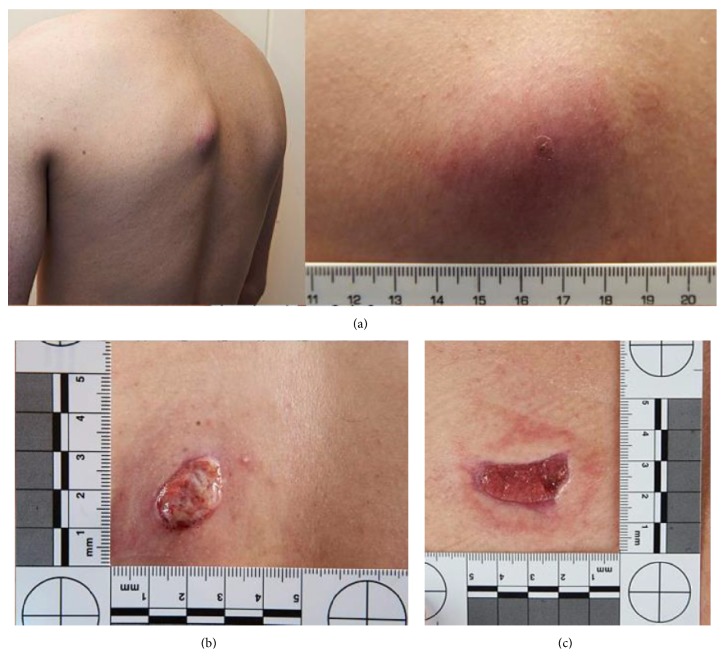
(a) Appearances of the closed abscess after one week of antibiotics (note central puncture site from previous diagnostic aspirate). (b) Proliferating granulation tissue, four weeks after top images were taken. (c) Healing ulcer, four months after the top images were taken.

One week later the patient's swelling had increased in size (Figure 1(a)). A second aspirate was performed and both this and the previous sample were then cultured on Sabouraud agar. This revealed a filamentous fungus that was later confirmed by the reference laboratory to be* Acremonium* spp. Ribosomal 16S had also been performed but did not identify a causative organism. Three weeks later the acremonium was reported to be sensitive to itraconazole, voriconazole, posaconazole, and amphotericin B but resistant to fluconazole.

Following confirmation that the infection was fungal in nature, itraconazole had been stopped and voriconazole commenced. Levels were taken at 8 and 30 days into treatment and found to be 3.91 and 3.97 mg/L, respectively, indicating adequate levels for treatment. A CT scan was performed to exclude the possibility that the infection was tracking from an internal source and a bronchoscopy with washing was performed to exclude a pulmonary origin for the infection. These investigations lead us to conclude that the infection was limited to the skin.

The closed abscess spontaneously discharged and granulation tissue proliferated through the exit site (Figure 1(b)). A short course of low dose prednisolone was used alongside voriconazole to help this resolve without surgical intervention. After 8 weeks of treatment with voriconazole, the patient was recommenced on itraconazole (at the increased dose of 200 mg twice daily). Eventually the granulation tissue resorbed and healthy skin started to close over the ulcerated area (Figure 1(c)).

## 3. Discussion

*Acremonium* spp. have been reported to cause pulmonary infection in children with CGD on two previous occasions [4, 5]. To our knowledge, this is the first instance where an adult with CGD has been infected with acremonium or where the skin has been the main site of infection.

*Acremonium* spp. are filamentous moulds which are widespread in environment materials such as soil or dead plant matter. They are a recognised but rare source of infection in humans. Cutaneous infections are one of the most common presentations in the immunocompetent host [6]. Mycetoma can form when localised trauma allows the fungus to penetrate the skin's barrier. In the developing world this would typically occur as a result of walking barefoot. However such infections are also recognised to occur without a history of trauma, as in this case. Invasive acremonium infections are more pertinent to the immunocompromised host, although the majority of reported cases relate to immunodeficiency that is secondary to medication, malignancy, and transplantation.

The available literature suggests that patients with CGD rarely develop infections due to acremonium. In the only reported cases of this occurring, the lung has been the primary site of infection. In Brazil, an infant was diagnosed with an acremonium lung infection after developing a hilar pneumonia that failed to respond to antibiotics [5]. Oral itraconazole (at 10 mg/kg/day) was used for treatment, with both clinical and radiological improvement being seen within 6 weeks. In a case report from 1984, a 15-year-old boy with CGD presented with suspected pneumonia and failed to respond to antibiotics [4]. He responded clinically but not radiologically to treatment with amphotericin and ketoconazole. This may account for why his treatment was continued for over 3 months.

When infections with moulds do occur in CGD patients, the mortality is high (16/49 cases, 32.7%) [3]. This case highlights the need for clinical immunologists to ensure that antifungal prophylaxis is maintained in patients with CGD. An increasing proportion of patients with CGD are being treated definitively with stem cell transplantation. Gene therapy is also a potential treatment for CGD patients and research into this technique is ongoing. The fact that breakthrough fungal infections can occur in adult life in untransplanted patients is a reminder of why parents are encouraged to choose transplantation for their children while they are young and free from infection.

Finally, this case highlights the importance of good communication between microbiology and immunology departments when sending samples from immunodeficient patients. Microbiology laboratories should be vigilant to the possibility of fungal and other fastidious infections when deciding how to process samples from immunodeficient patients.

## 4. Conclusion

Acremonium is a rare cause of infection, with patterns of infection being different between immunocompetent and immunocompromised hosts. While patients with CGD are recognised to be at increased risk of fungal infections, infections with acremonium are highly unusual and to our knowledge a case of acremonium causing a cutaneous infection has never been reported in CGD. The fungus was sensitive to itraconazole, yet the infection occurred while the patient was taking itraconazole prophylaxis. The infection improved following 8 weeks of treatment with voriconazole, and no recurrence has been observed in the subsequent 6 months.
